# 

**Published:** 1895-04-06

**Authors:** 


### The Hospital

AN INSTITUTIONAL JOURNAL OF

THE MEDICAL SCIENCES AND HOSPITAL ADMINISTRATION,

INCLUDING

A SPECIAL SUPPLEMENT FOR NURSES.

EDITED BY

HENRY C. BURDETT.

IN TWO VOLUMES ANNUALLY.

**VOLUME XVIII.**

APRIL TO SEPTEMBER, 1895.

SURGEON GENL'S OFFICE
152422
LIBRARY.

London:
PUBLISHED BY THE SCIENTIFIC PRESS (LIMITED), AT THEIR OFFICE, 428, STRAND, W.C.

MDCCCXCV.

W1 NO69X

### INDEX TO VOL. XVIII., 1895.

*** Articles under the general heading *Medical Progress and Hospital Clinics* are distinguished by the initial (C.) for *Hospital Clinics*, and by (P.) for the rest of the series. (W.) distinguishes the *Institutional Workshop* articles, and (A.H.) the notes under the general title *Around the Hospitals*.

Abdominal Surgery (P.), 344
Abdominal Wall, The Incision in the (Appendicitis) (P.), 133, *and see p.* 344
Abscess, Cerebral (P.), 308, 448
Absorbable Plates, Use of in Visceral Approximation (P.), 65
Adelaide Hospital, Adelaide, The (W.), 119
Adulterated Milk at Public Institutions, 23
Albuminuria (P.), 83
Alcohol in Workhouses, 163
Alcohol in Health and Disease (C.), 341
Ambulance in Peace and War (W.), 275
Ambulance System for Paris, An, 59
American Hospital, An (Poem), 34
Amyotrophic Lateral Sclerosis (P.), 149
Anastomosis, Intestinal (C.), 95, (P.) 433
Aneurism (P.), 28
Aneurism, The Symptoms of, 425
Aneurism, Unsuspected Thoracic (C.), 327
Aneurisms, The Causation of, 39
Aneurisms, The Classification of, 161, 231
Aneurisms, The Life-History of, 300
Angio-Neurotic Œdema (P.), 364
Annual Meeting, The, 5
Anterior Poliomyelitis (P.), 150
Antimony in Lung Disease (P.), 45
Antiseptic Surgery *See p.* 248
Antiseptics (P.), 185
Antiseptics, Internal (C.), 166
Anti-toxin. *See p.* 346
Anti-toxin for Snake Bite, 181
Anti-toxin, A Patent for, 23
Anus, Artificial (P.), 83, 434
Appendicitis (P.), 99, 412
Appendicitis Obliterans (P.), 99
Appendicitis, Recurrent (P.), 133
Appendix, Perforation of the, (P.), 99
Appointments. *See The Student of Medicine*
Approximation, Intestinal (P.), 64, 65
Are Women Worse than Men? 180
Around the Hospitals (A. H.) (*See also under the names of the various hospitals*), 86, 118, 155, 191, 277, 296, 350
Arthritis, Rheumatoid (P.), 342
Articular Origin, Lumbago and Acute Torticollis of (P.), 115
Ascites, Tubercular (P.), 270
Asthma (P.), 237
Ataxy, Locomotor (P.), 149
Attractions of Medicine, The, 387
Bacteriology, Value of, in Abdominal Surgery (P.), 344
Baldness, Cause of. *See p.* 380
Balfour (Mr.), on Medicine, 123
Base of the Skull (Fractures) (C.), 287
Beef-tea: Its Body and Soul, 234
Benzine Poisoning (P.), 240
Berthon Portable Hospitals (W.), 295
Bicycles and Tricycles, A Surgeon on, 373
"Biliousness," 59
Birmingham Hospital Saturday Fund, The, 145
Birmingham Throat and Ear Hospital, 355
Bladder, Cystitis and Tubercle of the (P.), 48
Bladder, Rupture of the (P.), 10
Blepharitis, Pyoctanin in Ciliary (P.), 373
Blood-letting Without Loss of Blood (C.), 61
Blood-vessels of Spinal Cord. *See p.* 149
Bombay Lying-in Hospital, 365
Bombay M.B., A, 93
Book World of Medicine, The (*Books, Magazines, and Pamphlets reviewed or noticed and articles relating to*):—
Beach. Mentally Enfeebled Children, 157
Berdoe. Microbes and Disease Demons, 385.
Billings and Hurd (introd. by Mitchell). Suggestions to Hospital and Asylum Visitors, 175
Bosanquet (Ed. by). Aspects of the Social Problem, 426
Brodie. Dissections Illustrated (Part IV.), 71
Burdett. Hospital and Charities Annual, 1895, 351
Cheadle. Artificial Feeding and Food Disorders of Infants, 87
Clark. Elementary Text-book of Anatomy, 17
Clodd. The Story of Primitive Man, 421
Collinson. Rainmaking and Sunshine, 333
Corfield. Dwelling Houses, 175
Cribb. *See Robinson*
Crookshank. The Prevention of Small-pox, 245
Davies. A Handbook of Hygiene, 157
Dolan. Our State Hospitals, 53
Dowkonnt. Murdered Millions, 216
Duhrssen (trans. by Taylor). Manual of Gynæcology, 279
Dutton. Food and Drink Rationally Discussed, 175
Francis. *See Small*
Fürbringer (trans. by Gilbert). Text-book of Diseases of the Kidneys and Urinary Organs, 193
Gilbert. *See Fürbringer*
Gimlette. Myxœdema and the Thyroid Gland, 421
Hall. Diseases of the Nose and Throat, 261
Horne. Guide to Whitby, 385
Book World of Medicine (*continued*)
Hurd. *See Billings*
Lindley. G.E.R. Tourist Guide, 279
Lindley. The Old Flemish Cities and the Ardennes, 245
Lindley. Walks in Belgium, 421
Lombroso. *See p.* 180
Magazines of the Month, 193
Maxwell. Drainage Work and Sanitary Fittings, 121
Medical Annual, The, 17
Mitchell. *See Billings*
Moullin. Enlargement of the Prostate, 87
Murray and Ravenstein. How to Live in Tropical Africa, 139
Murrell. Clinical Lectures on the Prevention of Consumption, 333
Nash. *See Small*
Nordau. Degeneration, 162
Nunn. Growing Children and Awkward Walking, 193
Pepper. Elements of Surgical Pathology, 121
Podmore. Apparitions and Thought Transference, 302
Rabogliati. Ovarian Neuralgia, 245
Rake. *See Schimmelbusch*
Ravenstein. *See Murray*
Reid. Housing the People, 266
Robinson and Cribb. Law and Chemistry of Food and Drugs, 455
Schimmelbusch (trans. by Rake). Aseptic Treatment of Wounds, 35
Scientific and Learned Societies' Year Book, 279
Small, Francis, and Nash. Anglo-Urdu Medical Handbook or Hindustani Guide, 139
Spencer. A New Method of Inhalation, 157
Taylor. *See Duhrssen*
Telford-Smith. Cases of Sporadic Cretinism Treated by Thyroid Extract, 279
Thompson. Reminiscences of Medical Missionary Work, 216
Tomlin. The Droitwich Brine Baths, 17
Vines. Student's Text-book of Botany, 385
Warner. Holiday Tramps Through Picturesque England and Wales, 157
Wiggin. Intestinal Anastomosis, 53
Williams. Diseases of the Breast, 71
Boscombe Hospital (W), 435
Brain Development, Partial Arrestments and Postponements of, 215, 283
Brain Surgery (P), 431, 448
Bread (Dietetics) (P), 63
Bright's Disease. *See p.* 81
Bristol Philosopher or What? A, 111
Bristowe, Dr. J. S., The Death of, 373
British Medical Association, The. Presidential and Other Addresses (C.), 305; Annual Museum (W.), 311, 330; Proceedings of Sections, 323
British Medical Association, The, and Antiseptic Surgery, 248, *and see p.* 353
British Medical Association, The, in London, 281
Buphthalmos (P.). *See p.* 346
Buried Alive, 427
Cab-call, The Moral of the, 111
Cæcum, Resection of the (P.), 83
Calcium Chloride in Lung Disease (P.), 45
Calomel and Potassium Bromide (P.), 240
Cambridge shows Oxford the Way, 20
Cancer (P.), 447
Cancer of the Colon (P.), 83
Cardiac Failure, Pain in (P.), 29
Cardiac Hypertrophy in Bright's Disease (P.), 271
Caravan Hospital. *See p.* 277
Carcinoma of the Stomach (P.), 379
Carotid Artery, Ligature of the Common (P.), 271
Cataract as it is met with in Ordinary Practice (C.), 113, 129
Cataract Extraction (P.), 274
Cavity Walls for Hospital Wards (W.), 52
Cerebral Abscess (P.), 308, 448
Cerebral Compression (P.), 239
Cerebral Hæmorrhage (P.). *See p.* 271
Cerebral Surgery (P.), 239, 257, 271, 308
Cerebral Tumours (P.), 289
Cerebro-spinal Meningitis (P.), 131
Cerebro-spinal Fluid, Drainage of the (P.), 271
Certificate Nuisance, The, 196
Cervical Canal (during Menstruation) (P.), 414
Charing Cross and King's College Hospitals, Position of (W.), 189
Charity and Brains, 251
Cheap Dispensing, 5
Chest, Congenital Deformity of the (P.), 393
Child-Life in France, 408
Child-Life in London, 426
Children, Diseases of (P.), 168, 184, 203
Chloralose (P.), 151
Chloroform Fatalities, 179, 249, 371, 441
Cholecystectomy (P.), 151
Cholelithiasis (P.), 150
Cholera (P.), 131
Ciliary Blepharitis (P.), 373
Ciliary Region and Lens, Penetrating Wounds of (P.), 273
Circulation, Physics of the (P.), 9
Circulatory System, Diseases of the (P.), 9, 28
Cirrhosis, Biliary, in Children (P.), 185
Clark, Sir A., The Memorial to, 424 (*and see p.* 405)
Clinical History, A Byzantine, 58
Clinical Research, 267
Clocks and Heart Disease, 388
Club-foot (C.), 26, 43
Congenital Treatment of (P.), 394
Paralytic (P.), 394
Coatbridge, A Hospital for, 409
Cold Applications in Lung Disease (P.), 46
Cold in the Head of Influenzal Origin, Causes, &c., of (C.), 80
Colon, Cancer of the (P.), 83
Conjunctivitis, Diphtheritic, and Anti-toxin (P.), 346
Construction Notes (W.), 34, 70, 156, 174, 226, 278, 349, 384, 420, 436, 453
Consumption Hospital, National (W.), 171
Consultants and General Practitioners. *See p.* 263
Cornea, Conical, 319
Cornea, Conjunctival Occlusion in Wounds of the (P.), 273
Cornea, Diseases of the, 21, 75, 105, 265, 319, 389
Cornea, Suture of the (P.), 274
Cornea, Vascularised (P.), 273
Corneal Affections, Phenate of Mercury in (P.), 273
Corneal Ulcers, Tincture of Iodine in (P.), 273
Correspondence (*Editor's Letter-Box*)
Cost of Patients in Fever Hospitals (W.), 15
Cranium, Notes on the Effect of Injuries to the Different Parts of the (C.), 219
Craniectomy in Microcephalic Idiots (P.), 272
Cremation on the Battlefield, 178
Criminology, 337
Cycle of Life, The, 318
Cyst, Pancreatic (P.), 170
Cystico-Lithectomy (P.), 151
Cystitis and Tubercle of the Bladder (P.), 48
Damaged Lives and Assurance Medicine, 74
Dermatology, Periscope of (P.), 363, 380
Desquamation after Scarlet Fever, 38
Devonshire Hospital and Buxton Bath Charity, The (A.H.), 118
Diabetes (P.), 98
Dietetics (P.) 62
Discredited Hospitals, 427
Disinfector, A Steam (W.), 153
Dismissal, Notice of (in the Case of Nurses), 437
Doctor and His Fees, A, 127
Doctor, The, and the Lawyer: An Ancient Conflict, 321
Doctor on Guard, The, 59
Doctor on Wheels, The, 391
Doctor or Schoolmaster? 199
Doctor or Spy? 145
Doctor's Twopence, The, 217
Doctor's Verdict, The, 357
Drink and Accidents, 1, 20
Dropsy, Renal (P.), 82
Drug and Chemical. &c., Trades Exhibition, 436
Drugs. *See New Appliances and Things Medical*
Drugs and Disease, 124
Drugs and Drink, 442
Drugs Many, Remedies Few, 317
Drunk or Sober? 354
Dundee Royal Infirmary (W.), 24
Dysmenorrhœa, Electricity in Cases of (P.), 414
Ear Disease in Childhood (P.), 203
Economics, A School of, 409
Ectopic Pregnancies, A New Classification of (C.), 375
Eczema (P.), 363
Editor's Letter-Box, 12, 84, 100, 138, 174, 192, 332, 403, 437, 454
"Egomania," 217
Egypt, A New Race in, 407
Election, An Old-fashioned (W.), 49
Electrical Treatment (in Gynæcology) (P.), 413
Embolism and Thrombosis of the Mesenteric Bloodvessels (P.), 344
English Doctors Abroad, 439
Epidemic Muscular Rheumatism (P.), 115
Epilepsy, Surgical Treatment of (P.), 448
Epileptics, Home for (W.), 293
Epithelioma of the Eye-lids, Methyl Blue in (P.), 274
Epsom College (*and see p.* 89) (W.), 259
Erythema Urticatum (P.), 203
Essex and Colchester General Hospital (A.H.), 155
Eucalyptus Treatment at Leicester, 285
Evolution in Surgery, 142
Example, A Good, 217
Exophthalmic Goitre, Salicylate of Soda in (P.), 274
Extirpation of the Liver, Partial (P.), 134
Eye, A Source of Infection of the (P.), 273
Eye Disease, Intravenous Mercurial Injections in (P.), 291
Eye Disease, Sub-conjunctival Injections in (P.), 291
Eyes, Artificial, Care of (P.), 273
Farmyard Pump, The, 77

Feeding of Infants, The (P.), 204
Fever Hospital, The Function of a (W.), 135
Fever Hospitals, Cost of Patients in (W.), 15
Filtration of Water, The Domestic (P.), 449
Fistula, Intestinal, and Artificial Anus (P.), 83
Fistulæ, Uretro Vaginal (P.), 414
Fixation of the Spine (P.), 362
Flat-foot (P.), 394
Floors. *See Hospital Floors*
Fossa Anterior—Middle—Posterior, Fractures of (C.), 287
Fountain Hospital, The, 339
Fractures and Dislocations (P.), 223, 309
Fractures of the Base of the Skull, Localising of (C.), 287
Fresh Air for Old Folks, 443
Friends of Man, The, 443
Friedrich's Disease (P.), 149
Frozen Milk, 199
Gall Stones, Operations for. *See* p. 134
Gastrectomy (P.), 117
Gastric Ulcer (P.), 117, 378, 446
Gastro-Anastomosis (P.), 379
Gastro-Enterostomy (P.), 117, 379
Gastropexy (P.), 117, 379
Gastro-plication or Stomach Reefing (P.), 117
Gastrotomy (P.), 378
Gastrotomy for Removal of Foreign Bodies (P.), 117
General Medical Council, The, 187
General Medical Council, The, and the Midwives Bill, 181
Geneva, The Canton Hospital at, 401
Genito-Urinary Surgery (P.), 10, 29, 47
Genius and Insanity, 162
German Hospitals, Progress in (W.), 435
Glaucoma, Infantile, Producing Buphthalmos (P.), 346
Goitre, Exophthalmic. *See* p. 274
Gout (P.), 238, 343, 375
Grantham Hospital, The (A. H.), 118
Graves' Disease (C.), 359
Gynæcology, Progress in (P.), 413
Hæmato-Porphyrin (P.), 82
Hampstead Home Hospital (A. H.), 296
Hands, The Training of the, 4
Head, Injuries of the (P.), 239, 431
Health and Athletics, 338
Health, The Standard of (P.), 204
Heart Disease, The Alleged Increase of, 232
Heart Disease, The Modern Treatment of, 159
Heart Disease, The Treatment of, 355
Heart Disease, Sudden Death in (P.), 28
Heart Disease, Functional (P.), 29
Heart Troubles at Halifax, 181
Heating and Ventilation (W.), 33, 51
Hereditary Disqualification for Marriage, 23
Heredity. *See* p. 126
Hernia, Strangulated Diaphragmatic (P.), 84
Hernia, Traumatic, of the Lachrymal Gland (P.), 346
Herpes Zoster (P.), 363
Hip, Congenital Displacement of the (P.), 394
"Hospital, The," Position of, 2
Hospital Administration (W.), 49, 101, 189, 275, 329, 417
Hospital Car (note), 60
Hospital Construction (W.), 13, 32, 67, 119, 171, 241, 347, 365, 435
Hospital Festivals and Meetings (W.), 15, 33, 52, 86, 102, 120, 136, 154, 173, 209, 242, 260, 295, 331
Hospital Floors (W.), 138, 172, 191
Hospital Letters (W.), 349
Hospital Problem in London, The, 213
Hospital Saturday Fund. *See page* 358
Hospital Saturday in London, 437
Hospital Sunday in London, 179
Hospital Sunday in London. A Brief History (W.), 205
Hospital Sunday 1895, Results of, 198
Hospital Sunday Fund (W.), 293, 304, 315
Hospital Sunday Fund (Metropolitan) Donations, 227, 244
Hospital Sunday Supplement, Our, 163
Hospital Sunday Supplement:—
*Proportion of Diseases to Population in London*, 9
*Diseases from which Londoners Suffer*, 10
*How Disease is Distributed throughout London*, 12
*Ways and Means*, 15
*Raising the Wind*, 16
*A Year's Work in the Hospitals and Medical Charities of London*, 17
*Our illustration*, 20
Hospitals and Honorary Medical Service, The Misuse of, 247, 262, 282
House Surgeon, How Not to Elect a (W.), 33, 51
How Disease is Spread, 369
Huxley's Aim in Life, 233
Hydatid Disease (P.), 134
Hydatids, Pelvic (P.), 345
Hyoscine, Unusual Effects of (P.), 11
Hypertrophy of the Prostate, Treatment of at Northampton (C.), 62
Hypochondrium, Right, Anatomy of the (P.), 134
Ileo-cæcal Region, Tumours of the (P.), 433
Indian Troops, Our, 303
Indurative Mediastino-Pericarditis (P.), 28
Infantile Scurvy (P.), 168
Infants, The Feeding of (P.), 204
Infective Diseases (P.), 131, 220
Inflammation, Suppurative, of the Joints in Children (C), 7
Insane. *See Story of the Insane*
Insanity in the Medical Profession, 303
Inspector "in excelsis," The, 370
Intestinal Anastomosis (C.), 95 (P.), 433
Intestinal Approximation by means of Murphy's Button (P.), 64
Intestinal Diseases (in Children), (P.), 184
Intestinal Disorders (in Children), (P.), 168
Intestinal Fistula and Artificial Anus (P.), 83
Intestinal Obstruction (P.), 65, 432
Intestinal Resection and Anastomosis (P.), 433
Intestines, Perforating Ulcer of the (Laparotomy), (P.), 64, 434
Intra-Laryngeal Applications (C.), 183
Intra-Thoracic Œsophagotomy (P.), 116
Intra-Uterine Medication (P.), 414
Intra-Venous Mercurial Injections (in Eye Disease) (P.), 291
Intubation of the Larynx (P.), 204
Intussusception (P.), 432
Isolation, A Practical Aspect of, 303
Isolation Hospital (Small) (W.), 277
Jejunostomy (P.), 434
"Jerry-built" Houses and Diphtheria, 357
Joints, Suppurative Inflammation of, in Children (C.), 7
Kidney Diseases (P.), 44, 97
Kidney, Palpation of the (P.), 82
Kidneys, Effect of Ether-anæsthesia on the (P.), 82
King's College and Charing Cross Hospitals, Position of (W.), 189
Labium, Varix of the (P.), 414
Lachrymal Gland, Traumatic Hernia of the (P.), 346
Laminectomy (P.), 361
Laminectomy in Spinal Caries (P.), 362
Lancaster Infirmary (A. H.), 419
Laparotomy for Perforating Ulcer of the Intestines (P.), 64
Laryngitis, Membranous or Croupous, in Infants (C.), 183
Larynx, Intubation of the (P.), 204
Leech Extract and Thrombosis (P.), 11
Leeds Hospital for Women and Children (A.H.), 350
Leprosy Fund, The, 39
Lesions, Traumatic Intra-abdominal (P.), 345
Literature for the Million, 110
Lithectomy. *See* p. 151
Liver, Partial Extirpation of the (P.), 134
Liver, Wounds of the (P.), 134
Liver and Gall-bladder, Surgery of the (P.), 134, 150
London Fever Hospital (A.H.), 86
London, The Medical Schools of, 395
London Must Give Degrees, 391
London Underground, 127
London Wants Students, 391
London's Mental Wreckage, 443
Lower Extremity, Fractures of the (P.), 309
Lumbago and Acute Torticollis of Articular Origin (P.), 115
Lung, Fibroid Diseases of the (P.), 307
Lung Disease, Cold Applications in (P.), 46
Lungs, Diseases of the (P.), 236
Lungs and Pleuræ, Diseases of the (P.), 45
Lupus (P.), 363
Lying-in Hospital, Bombay (W.), 365
Lymph, Formation of (P.), 29
Malaria (P.), 131
Manchester Royal Infirmary (W.), 13
Massage. *See* p. 201
Massage and Mobilisation in Fractures (P.), 223
Mastoid Disease (P.), 448
Measles. Should Measles be Notified? 163
Measles, The Notification of, 233
Mediastino-Pericarditis, Indurative (P.), 28
Medical Defence and Protection, 233
Medical Profession, The (A Sermon), 198
Medical Politicians, 267
Medical Politics in Yorkshire, 299
Medical Schools of London, The, 395
Medical Study, The Commencement of, 390
Medico-Psychology and Psychiatry. *See Modern Meetings. See Hospital Festivals and Meetings*
Memorial, A Mismanaged, 405, *and see* p. 424
Meningitis, Cerebro-Spinal (P.), 131
Meningitis, Tubercular (P.), 269
Menstruation, Painful, Certain Cases of (C.), 129
Mento-posterior Impaction, Case of (C.), 411
Metatarsalgia. Morton's Disease (P.), 394
Metric System, The, 256
Metropolitan Hospital, The, 162
Metropolitan Hospital Sunday Fund: Particulars of Awards, 315
Metrorrhagia (P.), 415
Middlesex Hospital: New Operating Theatre (W.), 348, 382
Midwives Bill, The, 145, *and see* p. 181
Midwives Bill, Some Points in the, 107
Milk (Dietetics) (P.), 62
Misuse of Hospitals, The, 247, 262, 282
Modern Medico-Psychology and Psychiatry, 3, 91, 143, 214, 283, 337
Modern Progress in Ophthalmic Medicine and Surgery, 21, 75, 125, 197, 265, 319
Modern Sociology, 4, 40, 76, 92, 105, 144, 250
Moral Mania, 320
Morals of a Man of Science, The, 321
Morphia in Gynæcological Practice, Dangers of (P.), 415
Morton's Disease, or Metatarsalgia (P.), 394
Murphy's Button. *See* p. 64
Muscular Rheumatism, Epidemic (P.), 115
Mushrooms and Manure Heaps, 5
Nævi (C.), 235
Nation, The, and its Paralytics and Epileptics, 55
National Hospital for Consumption, Ireland (W.), 171
National Society for the Employment of Epileptics Home (W.), 293
Nervous Children, The Education of, 56
Nervous Diseases (P.), 149
Nervous Diseases (in Children) (P.), 184
Nervous Diseases and Diseases of Development, 143
New Appliances and Things Medical, 11, 65, 84, 100, 152, 170, 186, 204, 224, 240, 258, 274, 292, 310, 381, 394, 416, 434, 450
Newtown Hospital (W.), 171
Notes and News, 6, 24, 43, 60, 78, 94, 112, 128, 146, 164, 182, 200, 218, 234, 252, 268, 286, 304, 322, 340, 358, 374, 392, 410, 428, 444
Notes and Queries, 120
Novel Reading. Does Novel Reading Prevent Marriage? 41
Nurses, their Uniform and the Public, 423
Ocular Headaches (P.), 274
Œdema, Angio-Neurotic (P.), 364
Œsophagitis Gangrenosa (P.), 116
Œsophagotomy, Intra-thoracic (P.), 116
Œsophagus, Cicatrical Stricture of the (P.), 377
Œsophagus, Diverticulum of the (P.), 117
Œsophagus, Foreign Bodies in the (P.), 377
Œsophagus and Stomach, Surgery of the (P.), 116, 377
Old Age Pensions, 250
On Sight-seeing, 440
"One Step" at the Tottenham Hospital, 41
One-and-a-Penny-Ha'penny, 372
Operating Theatre, Middlesex Hospital (W.), 348, 382
Ophthalmia, Purulent, Formol in (P.), 274
Ophthalmic Medicine and Surgery, Modern Progress in, 21, 75, 125, 197, 265, 319
Ophthalmology, Progress in (P.), 273, 291, 346
Opium in India, 74
Opium-fed Infants, 127
Optic Neuritis in Ozæna (P.), 273
Orthopædic Surgery (P.), 393
Oxford and its Infirmary, 93
Palpation of the Kidney (P.), 82
Panacea in English Medicine, The, 22
Pancreas. *See Spleen.*
Pancreas, Inflammatory Affections of the (P.), 169
Pancreatic Cyst (P.), 170
Panopthalmitis, Evacuation of the Eyeball in (P.), 273
Papain (P.), 30
Paracentesis of the Pericardium (P.), 29
Paralysis Agitans (P.), 150
Paralytics and Epileptics, The Nation and its, 55
Parasitic Diseases (Dermatology) (P.), 364
Parietal Incision, The (Abdominal Surgery) (P.), 344
Parsee Lying-in Hospital, Bombay (W.), 365
Pay Hospitals: Fiat experimentum, 300
Pay Hospitals or Medical Hotels, 37
Pay Wards, The Outcry Against, 77
Pelvic Hydatids (P.), 345
Peptonuria (P.), 83
Percussion Notes (Fracture of the Skull) (C.), 288
Perforating Ulcer of the Intestines (Laparotomy) (P.), 64, 434
Pericarditis, Indurative Mediastino (P.), 28
Pericardium, Paracentesis of the (P.), 29
Perigastric Adhesions (P.), 379
Peritonitis, Septic General (P.), 344
Perityphlitis (C.), 429
Perth City and County Infirmary (A.H.), 118
Phthisis (P.), 255
Physiology of Recreation, The, 335
Pleuræ (*Lungs*)
Pleurisy (P.), 237
Pneumonia (P.), 45, 236
Poets, The Scientific Manufacture of, 267
Poison Bottles (W.), 366
Poisonous Foods (Dietetics) (P.), 63
Police, The Clothing of the, 373
Poliomyelitis, Anterior (P.), 150
Polygamy and Progress, 427
Polymyositis (P.), 115
Poplar Hospital for Accidents, The (W.), 67
Poplar Hospital in Danger, 285
Potable Water, The Storage of, 214
Potassium Bromide and Calomel (P.), 240
Pott's Disease, The Treatment of (C.), 253; (P.), 262
Practical Departments (W.), 33, 51, 69, 85, 136, 153, 172, 191, 244, 277, 294, 348, 366, 382, 402, 418, 436, 452
Practical Points (W.), 15, 52, 86, 138, 437
Pregnancies, Ectopic (C.), 375
Preputial Malformation in the Newly Born (C.), 411
Private Hospitals in Dublin, 77
Psoriasis (P.), 363
Public Slaughter-houses and Sound Meat, 339
Puerperal Fever, The Prevention of (C.), 25
Pulmonary Congestion and its Treatment, 57, 109
Pyloroplasty (P.), 117
Pylorus, Non-malignant Stricture of the (P.), 378
Queen's Hospital, Birmingham, The (A.H.), 191

152422

Queen's Jubilee Hospital, The (W.), 329
Radcliffe Infirmary, Oxford, 339
Radius and Ulna, Case of Fracture of the (C.), 445
Raynaud's Disease (P.), 10
Recent Science, 108
Renal Diseases (P.), 204
Renal Dropsy (P.), 82
Rentoul's (Dr.) Rhapsody, 230
Research in Medicine, Some Aspects of, 160
Resection in Stricture of the Urethra (C.), 359
Respiration of Plants, The, 406
Respiratory System, Diseases of the (P.), 255, 269, 306
Respiratory System, Minor Affections of (P.), 308
Respiratory System, Pathology of (P.), 306, 308
Resuscitation of Still-born Infants, The (P.), 203
Rheumatic Affections (P.), 115
Rheumatism (P.), 115
Rheumatism, Acute (P.), 342
Rheumatism and Gout (P.), 342, 375
Rheumatism, Epidemic Muscular (P.), 115
Rheumatoid Arthritis (P.), 343
Richmond, Royal Hospital (A.H.), 419
Royal Medical Benevolent College, The, 89, *and see p.* 259
Royal Surrey County Hospital (A.H.), 155
Rupture of the Bladder (P.), 10
Rupture of the Urethra (P.), 11
Rupture of the Vas deferens (P.), 48
St. George-the-Martyr, A New Lung for, 251
St. Mary's Hospital (A.H.), 277
Samaritan Free Hospital (W.), 31
Scarlet Fever, Desquamation After, 38
Schoolmaster or the Doctor? The, 141
Schoolroom, Air of the, 336
Sciatica, An Early Symptom in Pott's Disease (P.), 362
Scientific Giants. Are Scientific Giants Extinct? 251
Sclerosis, Amyotrophic Lateral (P.), 149
Sclerotomy (Posterior) and Sclerotomy in Glaucoma (P.), 346
Scoliosis (P.), 393
Scoliosis, Clinical Aspects of (C.), 147, 165
Scoliosis, Pathological Anatomy of (P.), 393
Scopolamine as a Mydriatic (P.), 274
Scourge of India, The, 90
Scraps and Gleanings, 16, 35, 70, 86, 106, 121, 138, 156, 174, 192, 226, 244, 261, 278, 297, 333, 350, 367, 384, 403, 420, 437, 455
Scurvy, Infantile (P.), 168
Self-help and the Heads of the Profession, 285
Serum Inoculation. *See p.* 222.
Serum Therapeutics at the B.M.A., 353
Shahzada, Visit of, to St. Thomas's Hospital, 402
Ship's Surgeon, The Powers of the, 409, 454
Sight-seeing, On, 440
Sinus Thrombosis (P.), 449
Snake Poisons and Antidotes, 229
Social Problems, 216, 266, 408, 426
Sociology, Modern, 4, 40, 76, 92, 105, 144, 250
Some Points in the Midwives Bill, 107
Souls, The Speech of, 302
Southampton Royal South Hants Infirmary, 451
Special Supplement, Distributing the (W.), 207
Spina bifida (P.), 377
Spinal Caries, Pott's Disease (P.), 362
Spinal Column, Syphilitic Disease in the (C), 114
Spinal Cord, *See p.* 149
Spinal Disease, Supports in (P), 393
Spinal Diseases, Relation of, to Spinal Cord (P), 149
Spine and Cord (Injuries, Surgery) (P), 361, 376
Spleen, Axial Rotation of the (P.), 169
Spleen, Gunshot Wounds of the (P.), 169
Spleen and Pancreas, Surgery of the (P.), 169
Splenectomy (P.), 169
Sprains and Fractures, &c. (C.), 201
Staff in Small Hospitals, The Number of the (W.), 15
Standard of Health, The (P.), 204
Still-born Infants, The Resuscitation of (P.), 203
Stomach, *See Œsophagus, Gastric, Gastro*
Stomach, Carcinoma of the (P), 379
Stomach, Diseases of the (C.), 446
Story of the Insane from Year to Year, The (W.), 14, 68, 154, 175, 190, 242, 276, 367, 383, 401, 418, 456
Strabismus, *See p.* 346
Strangulation of the Testicle (P.), 48
Street Collections for Charities, 406
Stricture of the Pylorus, Non-Malignant (P.), 378
Stricture of the Urethra (P.), 47, 359
Stricture (Cicatrical) of the Œsophagus (P.), 377
Student of Medicine, The (*Appointments, Vacancies, Notes*), 17, 53, 71, 87, 106, 122, 139, 157, 175, 193, 228, 245, 261, 279, 297, 316, 333, 352, 367, 385, 404, 421, 438, 456
Study of Man, The, 407
Stuttering, The Physiology and Cure of, 195
Subconjunctival Injections in Eye Disease (P.), 291
Sugar (Dietetics) (P.), 63.
Summer Session, The, 20
Suppurative Inflammation of the Joints in Children (C), 7
Surgery, Evolution in, 142
Surgery, Abdominal (P.), 344
Surgery, Brain (P.), 431, 448
Surgery, Cerebral (P.), 239, 257, 271, 308
Surgery, Genito-Urinary (P.), 11, 29, 47
Surgery of the Intestines (P.), 64, 83, 432
Surgery of the Liver and Gall-bladder (P.), 134, 150
Surgery of the Œsophagus and Stomach (P.), 116, 377
Surgery, Orthopædic (P.), 393
Surgery of the Spine and Cord (P.), 361, 376
Surgery of the Spleen and Pancreas (P.), 169
Surgery of the Ureter (P.), 29
Surgery of the Vermiform Appendix (P.), 99, 133
Sydney Hospital (A.H.), 296
Sydney Hospital for Sick Children (A.H.), 278
Sydney, Prince Alfred Hospital (A.H.), 419
Symblepharon, Skin-grafting for (P.), 273
Syphilis, Latent Hereditary (P.), 203
Syringomelia (P.), 150
Talipes (P.), Calcareus, 27; Equinus, 26; Varus 27; Valgus, 27
Taxes or Tariffs? 40
Tenotomy of the Superior or Inferior Recta (P.), 346
Testicle, Strangulation of the (P.), 48
Then and Now in the Village, 93
Therapeutic Notes (P.), 11, 30, 151, 240
Thoracic Aneurism, Unsuspected (C.), 327
Throat Hospital, Golden Square, The, 321
Thrombosis. *See p.* 344
Thrombosis and Leech Extract (P.), 11
Thrombosis, Sinus (P.), 449
Thurso, District Hospital for (W.), 32
Thyroid Feeding (Dermatology) (P.), 380
Thyroid Gland, Physiology of the (P.), 360
Toothache Benefactor, A, 111
Toronto General Hospital (W.), 417
Torticollis, Spasmodic (C.), 269; Acute (P.), 115
Tottenham Hospital Dispute, The, 357, *and see p.* 41
Traumatic Hernia of the Lachrymal Gland (P.), 346
Traumatic Intra-Abdominal Lesions (P.), 345
Traumatic Stricture of the Urethra (C.), 359
Tubercle of the Bladder (P.), 48
Tubercle, Ætiology of the (P.), 270
Tubercular Ascites (P.), 269
Tubercular Meningitis (P.), 269
Tuberculosis, The Royal Commission on (C.), 17[illegible]
Tumours (P.), 364
Tumours, Cerebral (P.), 289
Typhoid (P.), 220
Typhoid Fever. *See Scourge of India*
Ulcer, Gastric (P.), 117, 378 446
Ulcer, Perforating, of the Intestines (P.), 64, 434
Ulster Eye, Ear, and Throat Hospital
Upper Extremity, Fracture of the (P.), 223
Uræmia (P.), 81
Ureter, Surgery of the (P.), 29
Urethra, Rupture of the (P.), 11
Urethra, Stricture of the (P.), 47
Urethra, Traumatic Stricture of the (C.), 359
Uretro-Vaginal Fistulæ (P.), 414
Urine Analysis (P.), 82
Urine, Secretion of, after Laparotomy (P.), 344
Urine Suppression (P.), 81
Uterine Appendages, Removal of (P.), 414
Uterine Curetting (P.), 415
Uterus, Diseases of (Due to Insufficient Food), (P.), 414
Vacancies. *The Student of Medicine*
Varix of the Labium (P.), 414
Vas deferens, Rupture of the (P.), 48
Ventilation of Hospitals. *See p.* 100
Ventilation and Heating (W.), 33, 51
Vermiform Appendix, Surgery of the (P.), 99, 113
Visceral Approximation (P.), 65
W. G. Grace Tribute, The, 199
Water, The Domestic Filtration of (P.), 449
Water, The Storage of Potable, 214
Weighing the Baby, 41
West Ham Hospital (W.), 347
Westminster Hospital, The (W.), 225, 259
Whooping-cough, The Treatment of (P.), 203
Within the Hospitals (W.), 31, 225, 381, 401, 435, 451
Wood Pavements and Disinfection, 264
Word to Living Londoners, A, 178
Wryneck, Operation for Spasmodic (P.), 272
York County Hospital (W.), 381

### THE SPECIAL SUPPLEMENT FOR NURSES.

Addenbrooke's Hospital, Cambridge, xiv
Alexandria and Cairo, Care of the Sick in, l, lvi, ci, cvii, cxvii, cxxxix
Alumnæ Associations, cli
American Letter, Our, xvi, xlviii, lxxiv, cv, cxxxviii, clxxvi
Anatomy and Surgery for Nurses, Elementary, iii, xi, xix, xxv, xxxi, xli, xlvii, lv, lxi, lxvii, lxxiii, lxxix, lxxxv, xciii, xcix, cv, cxi, cxxi
Appointments, viii, xiii, xix, xxv, xxxvii xli, lii, lvi, lxvii, lxxvi, lxxix, lxxxvi, xciv, cv, cxxv, cxxxiv, cxl, cxlix, clxii, clxviii, clxxvi
Appointments, Minor, xxi, xxviii, xlviii, lxxvi, lxxix, ci, cxxxviii, clxii, clxviii, clxxvi
Asylum [illegible]ews and Nursing, v, lxx
Book World for Women and Nurses, lviii, lxxxii, xcvi, cvii, cxviii, clii, clviii, clxiv, clxxvi
Burdett's Official Nursing Directory, xiv
Cairo. *See Alexandria*
Canada. A Nurse's Winter in, cxlvi
Choice of Pupils for Professional Training, On the, clvii
Crewe, A New Hospital for, lxx
Death in our Ranks, xli, lxii, lxxx
Dress and Uniforms, xv, xlii, lxxv
Dundee Royal Infirmary, xlvii, cxliii
Dutch News, xcvi
Everybody's Opinion, viii, xvi, xxvii, xxxvii, xliii, li, lvii, lxiv, lxix, lxxxi, lxxxix, ci, cvii, cxvii, cxxvi, cxxxiii, cxxxix, cxlv, cl, clxix, clxxv
Explanation, An, xcvi
Flat-foot Occurring in Nurses, cxvi, cxliv
French Schools for Trained Nurses, xxxiv, cxv, clxii
Friedenheim, xcvi
Friend, not Busybody, cxiii
Geneva, The Congress at, lxx
Holidays and Health, lxxxviii, xcv, cii, cxv, cxxxiii, cxlv
Holloway College, xcvi
The Hospital Convalescent Fund, cxxxi, cxxxvii, clviii, clxiv, clxviii, clxxiii
Hospital Secretaries at a Discount, xliii
Hospital Ship, A, cxxxiv
Hospital Sunday, lxxx
Indian Nursing Service Applications, lvii
Invalid Children's Aid Association, xxxiii
Invalids at Netley, xii
Junius S. Morgan Benevolent Fund, vii
Lady Dufferin's Fund, lxxxvii
Lahore Durbar of 1894, The Great, xxxv
Legacy, A Handsome, lxxxix
Letters from Upper Burma, xxxiii, l
Lewes District Nurses Fund, xlvii
Macclesfield Infirmary, lxxiv
Magazines for the Month, xxii
Massage, A Paris School of, lxxxvi
Medical Stores for Madagascar, xxv
Medico-Psychological Association, lxxx
Midwives, Registration of, xxxvii, cxiii
Murree, Jottings from, cxl
National Health Society, cxi
National Society for the Prevention of Cruelty to Children, lxxx
Netley, Volunteer Medical Staff Corps at, xxxviii
New South Wales, cxv
New York Training School for Nurses, Blackwell's Island, clxviii clxxiv
News from the Nursing World, i, ix, xvii, xxiii, xxix, xxxix, xlv, liii, lix, lxv, lxxi, lxxvii, lxxxiii, xci, xcvii, ciii, cix, cxix, cxxix, cxxxv, cxli, cxlvii, cliii, clix, clxv, clxxi
Notes and Queries, iv, xiv, xxxvi, xliv, lii, lvii, lxii, lxix, lxxiv, lxxxi, lxxxix, xciii, cii, cvii, cxii, cxxi, cxxxiv, cxxxix, cxlvi, cl, clxiv, clxx clxxvi
Notes from Germany, lxxxix
Notes from Ireland, lxiii
Notes from Victoria, xxi
Novelties for Nurses, viii, xxii, xxviii, xxxv, xliii, lvi, lxxxii, lxxxix, ci, cxiv, clvi, clxi, clxix
Nurse at Aden, A, xxxii
Nurses. Wanted—Nurses, clv
Nurses at Bradford Infirmary, lxxxvi
Nurses, Boston Training School for, lxviii
Nurses, Concerning, cxxxviii
Nurses' Co-operation. A Great Movement, vi, xiii
Nurses in the U.S.A., lxxxviii
Nurses on the Labrador, lxxxviii
Nurses' Pension Fund for Sweden, A, xliii
Nurses, Trained, at Bradford, cxxxiv
Nursing in America, xx, xxvi, xxxii, xciv
Nursing in France, iv
Nursing in Germany, xxi
Nursing in Japan, cxii
Nursing in Military Hospitals, lxx
Nursing in Rome, clvi, clxxiv
Nursing Association, Metropolitan, xlviii
Nursing, District, at Worthing, xii
Nursing, Hints for Home, cxliv
Nursing of Mental Cases, Notes on the Private, c
Nursing World, xxxiii
Ockley System, The, clxiii
Our Princess's Garden Party, cxxxi
Our Princess's Letter. cxxxvii
Paddington Green Children's Hospital, xcv
Pension Fund. *See Royal.*
Physiology for Nurses, Elementary, cxxxi, cxxxvii, cxliii, cxlix, clv, clxi, clxvii, clxxiii
Presentations, lxiv, ci, cv
Princess's Colours, cxxv
Queen Victoria's Jubilee Institute, lvi, xcv
Rational Dress in Russia, cxliv
Reading to the Sick, For, xxi, lviii, cxvi, cxl, clxx
Reminiscences of "First Aid," clvi
Royal British Nurses' Association, xxvii, lxxx, lxxxvi, xcvi, cii, cvii, clxxiv
Royal National Pension Fund for Nurses, lxix, lxxxv, cxiii, cxxii
St. Olave's Union Infirmary, clxiii
St. Peter's Society to Meiringen, With, lii
School for the Blind at York (Poem), lxxvi
Small-pox in the Desert—A Nurse's Experience, lxiv
Society of Trained Masseuses, The, lxvii
Training for Lecturers, cxxxi
Wants and Workers, vi, lxxxi, ci, cv, clii, clxix
Where to Go, xii, xxii, xxvii, xxxvi, li, lvii, lxiii, lxxv, lxxxi, lxxxviii, xciv, cv, cxxv, cxxxi, clxx, clxxiii
Workhouse Infirmary Nursing Association, lxii

Printed by W. H. and L. Collingridge, "City Press," 148 and 149, Aldersgate Street, London, E.C.

**Notices of Births, Deaths, and Marriages** are inserted in this column at a charge of 2s. 6d. for an announcement not exceeding four lines.

**Notice to Advertisers and Subscribers.**

All Advertisements, Orders for Copies of The Hospital and Business Communications should be addressed to **The Manager, NOT TO THE EDITOR,** The Hospital, 428, Strand, London, W.C.

The Cost of Subscription, post free, is as follows:—Per week, 2½d.; per quarter, 2s. 8d.; per half-year, 5s. 3d.; per annum, 10s. 6d. Foreign:—Per Annum, 15s. 2d.; payable, in all cases, in advance.

### NOTICE TO CORRESPONDENTS.

All MS., Letters, Books for Review, and other matters intended for the Editor should be addressed The Editor, The Lodge, Porchester Square, London, W.

The Editor cannot undertake to return rejected MS., even when accompanied by stamped directed envelope.

**"Burdett's Hospital Annual," 1895,**

Ready Immediately.

Sixth year of publication. About 1000 pp. Scarlet cloth, gilt lettered. Price 5s. A most complete guide to British, Colonial, Indian, and American Hospitals, Dispensaries, Nursing and Convalescent Institutions, and Asylums. A few copies of the "Annual" for 1894 may still be obtained on application to the Publishers, The Scientific Press (Limited), 428, Strand, London, W.C.

**NOTICE.—The Index for Vol. XVII (ending March 30th, 1895) will be on sale at the Office of this Journal in a few days, Price 2½d.. post free.**

**IMPORTANT NOTICE.**

**On account of the Easter Holidays, we shall go to press with our issue of the 13th instant on TUESDAY NIGHT, the 9th INST. Advertisements intended for insertion in this issue should therefore reach us BY NOON ON TUESDAY, the 9th inst.**

### Notes and News.

### Scraps and Gleanings.

The Clothworkers' Company have given a donation of £70 to the North-Eastern Hospital for Children in Shoreditch.

As a contribution to the special fund for opening the closed wards at St. Thomas's Hospital, Mrs. A. Hall has given £1,000 in memory of her late husband.

The sum of £525 has been granted by the Merchant Taylors' Company towards the St. Thomas's Hospital Special Appeal Fund, to be paid in annual instalments of 100 guineas.

A concert was given at the Queen's Hall last Saturday on behalf of the maintenance fund of the Morley House Oaxton wing in connection with the Morley House Seaside Convalescent Home at Dover.

The London and South-Western Railway has contributed £105 to the St. Thomas's Hospital Special Appeal Fund for opening closed wards. The annual subscription has also been increased from £15 to £26 5s.

A school of medicine for women is shortly to be established at St. Petersburg. A Russian lady has undertaken to furnish the annual sum of 12,000 roubles for 10 years, on condition that the school is opened in 1895.

The Hospital Saturday and Sunday Association of Baltimore has issued its thirteenth annual report, showing that in 1894 the collection amounted to about £384—to be divided between 15 hospitals and homes.

A scheme is on foot for the endowment, by subscription, of a cot at the Hospital for Sick Children, Great Ormond Street, as a tribute to the memory of the late Mr. Corney Grain. The sum required is about £1,000.

The current number of "Pears' Pictorial" (No. 7) contains an illustrated review of Thomas Rowlandson and his works, with interesting reproductions of some of his pictures and an account of his life and labours.

The proposal to open up to vehicular traffic the Middle Meadow Walk at Edinburgh has met with some protest on account of the noise reaching the medical wards of the Royal Infirmary which are on that side of the building.

The new wing to the Swindon Victoria Hospital will shortly be ready for occupation. The expenses of maintenance will then be considerably increased, in view of which the committee are urgently pleading for contributions.

The governors of the St. Mark's Ophthalmic Hospital at Dublin state that their banking account is still largely overdrawn, and earnestly appeal to the public for aid to enable them to carry on the good work of the hospital efficiently.

A sum of £8,000, the results of the Donnybrook Fair Bazaar, and generous gifts by individuals, is at the disposal of the Cork committee for the building of an eye, ear, and throat hospital in the city. The site is still under discussion.

The proposal to erect an infectious diseases hospital for Crieff is now under discussion. It is estimated that a suitable building comprisind four wards of two beds each, and two wards with one bed each, could be erected for about £800 or £1,000.

The annual report of the York County Hospital states that the institution has suffered financially from the trade depression during the previous year. There was, however, an increase of £31 in the amount received from workmen's collections.

A successful entertainment was recently held at the Royalty Theatre in aid of the building fund of the Throat Hospital, Golden Square. The comedietta of "Old Cronies" was followed by a new and original farce called "For Family and Fame; or, the Sea and its Dead."

It is intended to connect the Lodge Road Hospital and the hospital in the Western Road at Birmingham by means of a bridge. The hospital prepared for the reception of small-pox patients will be opened as soon as the requisite water supply can be obtained from the mains.

At the recent annual meeting of the subscribers to the Bath Homœopathic Hospital it was stated that the number of out-patients receiving relief from the hospital had amounted to 7,000 in the course of the last year, but notwithstanding this the accounts showed a smaller deficit than in some former years.

An action has been brought to restrain the Guildford, Godalming, and Woking Joint Hospital Board from erecting a small-pox hospital on Whitemoor Common. The plaintiffs, "persons interested in neighbouring property," contend that the site is unsuitable, and that the proposed hospital would be a source of danger.

It was stated at the late annual meeting of the Monkstown Hospital that £800 (Miss Cannon's bequest) had been transferred to the Nurses' Pension Fund. Votes of thanks were passed to the Monkstown Football Club, and Dalkey, Kelleney, and Glengeary Chrysanthemum Society, for their contributions to the funds of the hospital.

The 29th annual meeting of the subscribers to the Bristol Hospital for Sick Women and Children has recently been held. The committee stated that the indoor staff of the Bristol Post Office have subscribed £25 during the year, and have intimated their intention to contribute a similar sum annually with a view of supporting a cot.

It was stated at the recent annual meeting of the Chesterfield and North Derbyshire Hospital and Dispensary that the institution has existed for the past year entirely out of its own revenue. The question having arisen as to the admittance of medical cases, it was decided for many reasons to continue admitting surgical cases alone.

At the annual general meeting of the governors of the Poplar Hospital for Accidents it was stated that the cost of the enlarged site, building the new and altering the old hospital, had amounted to £30,918. The hospital is, however, entirely out of debt. The working men of the district gave and collected £649 for the institution during the past year.

The diminution in the amount of the subscription list to the Birmingham General Hospital has continued during the last year. The amount received from the musical festival was £4,500, being £500 less than in 1891. The New General Hospital Building Committee estimate the approximate cost of the building, site, and equipment at about £200,000. Towards this, subscriptions amounting to £99,457 have been promised.

### THE STUDENT OF MEDICINE.

### APPOINTMENTS.

S. R. Alexander, M.D.Lond., M.R.C.S.Eng., appointed Medical Officer for the First District of the Faversham Union. M. R. J. Behrendt, L.R.C.P., L.R.C.S.Edin., appointed Medical Officer of Health to the Brumby and Frodingham Urban District Council. E. Bromet, M.A., M.R.C.S., L.R.C.P., appointed House Surgeon to the West London Hospital, Hammersmith. C. Brooks, M.R.C.S., L.R.C.P.Lond., appointed Medical Officer for the Gerrard's Cross District of the Eton Union. E. C. Bury, M.D. St.And., M.R.C.S.Eng., appointed Medical Officer of Health to the Wisbech District Council. A. Cutfield, B.A.Cantab., M.R.C.S.Eng., appointed Medical Officer to the Man of Ross Oddfellows' M.U. W. H. Date, M.R.C.S.Eng., L.S.A., appointed Medical Officer of Health to the Wellington Rural District Council. G. H. Edington, M.B.Glasg., late R.M.O. Glasgow Hospital for Sick Children, appointed Surgeon to Glasgow Central Dispensary. F. A. M. Flegg, L.R.C.P.Lond., M.R.C.S.Eng., L.S.A., appointed House Surgeon to the Seamen's Infirmary and General Hospital, Ramsgate, and Visiting Surgeon to the Ramsgate and St. Lawrence Royal Dispensary. G. G. Fleming, M.D., appointed Medical Officer for the First District of the Ely Union. E. L. Gault, M.A., M.B., B.S.Melb., appointed Ophthalmic Surgeon to the Alfred Hospital, Melbourne. T. H. Gibson, M.B., C.M.Edin. appointed Police Surgeon for Kirkby Stephen. L. J. Godson, M.R.C.S., L.R.C.P., appointed Senior House Surgeon to the Infirmary, Shrewsbury. F. W. Gunn, M.D.Durh., M.R.C.S., L.R.C.P.Lond., appointed Medical Officer for the Fourth District of the Morpeth Union. J. A. Jones, L.R.C.P. Lond., M.R.C.S.Eng., appointed Medical Officer of Health for the Borough of Aberavon. Dr. R. Mitchell, appointed Medical Officer for the Hooton Pagnal District of the Doncaster Union. Mary C. Murdoch, L.R.C.P., L.R.C.S.Edin., L.F.P.S.Glasg., appointed Assistant Medical Officer to the North-Eastern Fever Hospital, Tottenham. Dr. O'Leary, appointed Medical Officer for the Courcey Dispensary District. O. F. Paget, M.B., B.C.Cantab., appointed Resident Medical Officer to the St. George's and St. James's Dispensary, W. F. M. Reynolds, M.B., C.M.Edin., appointed Medical Officer for the Newton Poppleford District of the St. Thomas's Union. W. A. G. Russell, M.A., M.B., C.M.Aber., appointed Medical Officer for the Wingage District of the Easington Union. C. E. Shaw, M.A., M.D., M.Ch.R.U.I., appointed Assistant Surgeon to the Belfast Ophthalmic Hospital. W. Taynton, L.R.C.P.Edin., M.R.C.S., appointed Medical Officer for the Linton District and the Workhouse of the Linton Union. A. C. Waters, M.B., B.S.Durh., M.R.C.S.Eng., appointed Medical Officer of Health for the Borough of Southend-on-Sea. Dr. Watts, appointed Medical Officer to the Ilchester Almshouse Charity Trust.

### VACANCIES.

The following vacancies are announced this week, the amount of salary in each case being given after the name of the institution:

### Resident Medical Officers.

County Asylum, Dorchester.—Second Assistant Medical Office; unmarried, and not over 30 years of age. Salary to commence £130 per annum, rising £10 yearly to £160. Applications to Dr. MacDonald, Medical Superintendent, by April 8th.

East London Hospital for Children, Glamis Road, Shadwell, E.—House Physician. Board, lodging, &c., provided; no salary. Applications to the Secretary by April 8th.

London County Asylum, Hanwell, W.—Fifth Assistant Medical Officer; doubly qualified, and between 23 and 30 years of age. Salary £120 per annum, rising by £5 a year to £150, with board, furnished apartments, and washing. Applications, on forms to be obtained at the office, to R. W. Partridge, Clerk to the Asylums Committee Asylums Committee Office, 21, Whitehall Place, S.W., by April 6th.

### THE STUDENT OF MEDICINE—*continued.*

Lincoln County Hospital.—House Surgeon. Under 30 years of age, and unmarried. Salary £100 per annum, with board, lodging, and washing. Applications to the Secretary by April 6th.

Royal Hospital for Sick Children, Glasgow,—House Surgeon and Assistant House Surgeon. Salary £60 per annum for the former and £36 for the latter, with board and washing in each case. Applications to M. P. Fraser, 91, West Regent Street, Glasgow, by April 6th.

West Riding Asylum, Menston, near Leeds.—Third Assistant Medical Officer. Salary to commence at £130 per annum, rising £10 annually to £160, with board, lodging, and apartments. Applications to the Medical Superintendent at the Asylum by April 6th.

### Non-Resident Medical Officers.

North-Eastern Hospital for Children, Hackney Road, Shoreditch, N.E. —Physician. Must be F. or M.R.C.P.Lond. Applications to T. Glenton-Kerr, Secretary, at the Office, 27, Clement's Lane, E.C., by April 16th.

London Hospital Medical College.—Assistant Demonstrator in Anatomy. Salary £90 per annum. Applications to the Warden by April 25th.

Royal College of Physicians.—Milroy Lecturer. Applications to the Registrar, Royal College of Physicians, Pall Mall East, S.W., by April 8th.

Tancred's Charities.—Two Studentships in Divinity at Christ College, and one Studentship in Physic at Gonville and Caius College, Cambridge. Each student will receive £50 a year and a share in the surplus rents and profits (the stipend not to exceed £100 a year); the student in Physic until admission to the degree of Bachelor of Medicine, and for three years afterwards, or for such shorter terms as may be determined by authority. Candidates must be natives of Great Britain, members of the Church of England, unmarried, and not below the age of 16 or above the age of 22. Forms of petition and all information may be obtained from Mr. George Edgar Frére, 28, Lincoln's Inn Fields, W.C., Clerk to the Tancred's Charities, to whom petitions must be sent by April 20th.

### Medical and Administrative Appointments.

**NORTH-EASTERN HOSPITAL for CHILDREN, HACKNEY ROAD, SHOREDITCH, N.E.**

T. GLENTON-KERR, Secretary.

Office, 27, Clement's Lane, E.C., March 18th, 1895. (41)

**THE HOSPITAL FOR SICK CHILDREN, GREAT ORMOND STREET, W.C.**

